# Radiofrequency Catheter Ablation of Supraventricular Tachycardia

**Published:** 2002-04-01

**Authors:** Hugh Calkins, VK Ajit Kumar, Johnson Francis

**Affiliations:** 1 Professor of Medicine Johns Hopkins Hospital, Baltimore, Maryland, USA; 2 Associate Professor of Cardiology, Sree Chitra Tirunal Institute of Medical Science and Technology, Trivandrum, India; 3 Associate Professor of Cardiology, Medical College Calicut, Kerala, India

**Keywords:** supraventricular tachycardia, atrioventricular nodal re-entry tachycardia, atrioventricular re-entry tachycardias

## Introduction

Supraventricular tachycardias are quite common in clinical practice. Medical treatment of supraventricular tachycardia often involves regular intake of drugs for several years. Problems of drug therapy include poor efficacy and bothersome side effects including proarrhythmia. This has lead to the development of non-pharmacological therapies. Arrhythmia surgery initially demonstrated that many types of supraventricular arrhythmias could be cured. However during the past decade arrhythmia surgery has been largely replaced by catheter ablation. Catheter ablation can be defined as the use of an electrode catheter to destroy small areas of myocardial tissue or conduction system, or both, that are critical to the initiation or maintenance of cardiac arrhythmias. Arrhythmias most likely to be amenable to cure with catheter ablation are those which have a focal origin or involve a narrow, anatomically defined isthmus [1]. This review aims to provide an update on the technique and results associated that can be achieved with catheter ablation of supraventricular tachycardias.

## Classification of Supraventricular Tachyarrhythmias

Supraventricular tachyarrhythmias have been classified into regular and irregular (atrial fibrillation) [2]. Atrial fibrillation is less amenable to cure with catheter ablation than are the regular forms of supraventricular tachyarrhythmias such as atrioventricular nodal reentrant tachycardia (AVNRT) and atrio-ventricular reciprocating tachycardia.(AVRT). Non-pharmacological treatment of atrial fibrillation has been reviewed recently [3]. Regular supraventricular tachycardias (SVT) can be cured by catheter ablation with high success rates (95-99%) and low complication rates (< 1%) [2]. Regular SVTs can be subdivided into:

1. Atrioventricular re-entry tachycardias (AVRT), using the ventricle as part of the circuit; these tachycardias are dependent on the presence of an accessory atrioventricular (AV) pathway.

2. Atrioventricular nodal re-entry tachycardia (AVNRT), where the re-entry circuit is within the AV node and the ventricle plays no part in maintaining the arrhythmia.

3. Atrial tachycardia, where the re-entry circuit does not involve any part of the AV junction e.g. atrial flutter, ectopic atrial tachycardia.

## Technique of Ablation

Catheter ablations are performed in cardiac electrophysiology laboratories specially equipped with recorders, programmed stimulators and ablators. Ever since 1989, radiofrequency current has been used for ablation, while earlier attempts were with direct current. The procedure is typically performed using conscious sedation. Two to five multipolar electrode catheters are inserted percutaneously under local anaesthesia into a femoral, brachial, subclavian, or internal jugular veins and positioned in the heart under fluoroscopic guidance. Each electrode catheter has four or more electrodes. The most distal electrode pair is usually used for pacing and the delivery of critically timed extra stimuli, while all of the electrodes are used to record electrograms from localised regions within the heart. Up to 50 W of radiofrequency energy is delivered for 30-60 seconds as a continuous, unmodulated, sinusoidal waveform with a frequency of approximately 500,000 Hz, between the 4 mm tip of a deflectable ablation catheter and a ground plate positioned on the patient's back or chest. The temperature of the ablation electrode can be monitored and the power output automatically adjusted to achieve a targeted electrode temperature of between 60-70°C. Knowledge of the electrode temperature at a particular ablation site is useful in determining whether an unsuccessful application of radiofrequency energy failed because of inaccurate mapping or inadequate heating [4]. If the failure was as a result of inadequate heating, additional applications of energy at the same site with improved catheter stability may succeed. Automatic adjustment of power output using closed loop temperature control has been shown to reduce the incidence of coagulum development. This reduces the number of times the catheter has to be withdrawn from the body to have a coagulum removed from the electrode tip. Thermal injury is the main mechanism of tissue destruction during radiofrequency catheter ablation. Elevation of tissue temperature results in desiccation and the denaturation of proteins, and coagulation of tissue and blood. Irreversible tissue injury occurs at temperatures above 50°C. When temperature reaches 100°C, plasma proteins denature to form a coagulum. The coagulum causes a sharp rise in the impedance and a corresponding fall in the current density, thereby limiting further lesion growth. Methods for improved cooling of the electrode have been developed to allow delivery of higher radiofrequency power without the formation of coagulum. These include the use of larger (8 mm) electrodes, which receive greater convective cooling by the blood, and saline irrigated electrode tips, in which the electrode is actively cooled. Biplane fluroscopic imaging gives a three dimensional orientation of catheter position, enabling better localization. Non fluroscopic imaging systems using magnetic fields and ultrasound are being developed. These modalities avoid the inherent risk of radiation exposure to the patient and the treating physician.

## Ablation of atrioventricular re-entry tachycardias (AVRT)

AVRT utilizes accessory pathways which are anomalous extra nodal connections between the epicardial surface of the atrium and ventricle along the atrioventricular groove. Accessory pathways can be classified based on their location along the mitral or tricuspid annulus, type of conduction (decremental or non-decremental), and whether they are capable of antegrade conduction, retrograde conduction, or both. Accessory pathways which are capable only of retrograde conduction are concealed while those capable of antegrade conduction are manifest, demonstrating pre-excitation on a standard ECG. AVRT is the most common arrhythmia in patients with a manifest accessory pathway, occurring in 75% of cases.

An electrophysiological study is performed to localise the accessory pathway and determine its conduction characteristics prior to ablation. Accurate localisation of an accessory pathway is critical to the success of catheter ablation procedures. Preliminary localisation of the accessory pathway can be determined based on the delta wave and QRS morphology on the surface ECG in patients with manifest pre-excitation. Mapping of concealed accessory pathways and more accurate localisation of manifest accessory pathways require analysis of the retrograde atrial activation sequence and/or antegrade ventricular activation sequence. Right sided and posteroseptal accessory pathways are typically localised and ablated using a steerable electrode catheter with a 4 mm distal electrode positioned along the tricuspid annulus or in the coronary sinus os from the inferior vena cava. The location of left sided accessory pathways can be determined using a multipolar electrode catheter positioned in the coronary sinus, which runs parallel to the left atrioventricular groove or with a steerable catheter positioned in the left atrium or ventricle. Once localised to a region of the heart, precise mapping and ablation is performed using a steerable 4 mm tipped electrode catheter positioned along the mitral annulus using either the transeptal or retrograde aortic approach. These two approaches for ablation of left sided accessory pathways are associated with a similar rate of success and incidence of complications [5]. The decision over which approach to employ is usually based on physician preference, although the transeptal approach may be preferable in the elderly and in young children. In rare instances, left sided accessory pathways can only be ablated via the coronary sinus.

Appropriate sites for radiofrequency energy delivery during ablation of manifest accessory pathways are characterised by early ventricular activation, the presence of an accessory pathway potential, and stability of the local electrogram [6]. Appropriate sites for energy delivery in patients with retrograde conduction accessory pathways mapped during ventricular pacing or orthodromic AVRT are characterised by continuous electrical activity, the presence of accessory pathway potential, and electrogram stability. Once an appropriate target site is identified, radiofrequency energy is delivered for 30-60 seconds with a target electrode temperature of 60-70°C. At successful ablation sites, interruption of conduction through the accessory pathway usually occurs within 10 seconds, and often within two seconds of the onset of radiofrequency energy delivery.

Efficacy of catheter ablation of accessory pathways varies from 89-99% [7-9]. The success rate is highest for left free wall accessory pathways and lowest for posteroseptal and right free wall accessory pathways. Recurrence of conduction occurs in approximately 7% [7-9].  Recurrence is more common following ablation of posteroseptal and right free wall pathways. Accessory pathways which recur can usually be successfully reablated. Complications associated with catheter ablation may result from vascular access (haematomas, deep venous thrombosis, perforation of the aorta, arteriovenous fistula, pneumothorax), catheter manipulation (valvar damage, microemboli, perforation of the coronary sinus or myocardial wall, coronary dissection and/or thrombosis), or delivery of radiofrequency energy (atrioventricular block, myocardial perforation, coronary artery spasm or occlusion, transient ischaemic attacks or cerebrovascular accidents). Complete heart block occurs in about 1% of patients, most commonly after ablation of septal and posteroseptal accessory pathways. The overall incidence of complications varies between 1-4%. Procedure related death is estimated to be less than 0.2% [9].

## Ablation of Atrioventricular Nodal Re-entrant tachycardia

Atrioventricular nodal re-entrant tachycardia (AVNRT) occurs in patients with dual AV nodal physiology. They have two functionally distinct pathways known as the slow and fast pathway. The slow pathway has a shorter refractory period. During the common form of AVNRT, an atrial premature beat travels down the slow pathway and gets conducted back by the fast pathway to depolarise the atrium. In the uncommon variety, the reverse pattern occurs. The fast pathway is located anteriorly along the septal portion of the tricuspid annulus, near the compact atrioventricular node, whereas the atrial insertion of the slow pathway is located more posteriorly along the tricuspid annulus, closer to the coronary sinus ostium.

AVNRT can be cured by ablating either the fast pathway or the slow pathway. However, it has clearly been shown that catheter ablation of the slow pathway using a "posterior approach" is associated with greater safety and efficacy. For this reason, the "anterior approach" is merely of historical interest. Fast pathway ablation is by an "anterior" approach. Catheter ablation is performed by locating an electrogram with a large His potential and then withdrawing the ablation catheter into the right atrium until the atrial signal is at least twice that of the ventricular signal (A:V ratio > 2) with a His potential no larger than 50 μV. Radiofrequency energy is then applied during sinus rhythm for 30-60 seconds while watching for prolongation in the PR interval. Energy delivery is immediately terminated if atrioventricular block occurs. Successful ablation of AVNRT is characterised by lengthening of the PR interval and the inability to induce the tachycardia. There is elimination or pronounced attenuation of retrograde conduction during ventricular pacing. The atrioventricular block cycle length and the atrioventricular node effective refractory period are not usually altered during ablation by the anterior approach. Anterior approach is successful in about 90% of cases. Inadvertent atrioventricular block occurs in approximately 7% of patients. Recurrence rate is about 9% [1].

Slow pathway ablation is by the "posterior" approach. The ablation catheter is directed into the right ventricle low near the posterior septum and is then withdrawn until an electrogram is recorded with a small atrial electrogram and a large ventricular electrogram (A:V ratio < 0.5). Specific ablation sites along the posterior portion of the tricuspid annulus can be selected based either on the appearance of the local atrial electrogram or based strictly on anatomic factors. While using the electrogram guided approach, fractionated atrial electrograms with a late "slow potential" are targeted. In the anatomic approach, the initial applications are delivered at the level of the coronary sinus ostium and subsequent applications at more superior sites. Junctional beats occurring during the application of radiofrequency energy are a marker for successful ablation. Successful ablation is characterised by an increase in the atrioventricular block cycle length and in the atrioventricular node effective refractory period and elimination of inducible AVNRT. The posterior approach for ablation of AVNRT is effective in greater than 95-97% of patients [9-11].  Atrioventricular block occurs in 0.5-1% and is the most common complication. Recurrence following successful ablation using the posterior approach is approximately 3%. Posterior approach is the preferred approach to ablation of AVNRT due to the higher efficacy, lower incidence of atrioventricular block and arrhythmia recurrence, and the greater likelihood of maintaining a normal PR interval during sinus rhythm.

## Atrial Tachycardias

Catheter ablation of sustained regular atrial tachycardias can also be performed with high safety and efficacy. Focal atrial tachycardia, whether reentrant, automatic, or triggered can be ablated with success rates greater than 90%. The site for ablation is generally chosen based on early activation. Typical atrial flutter is also amenable to cure with catheter ablation. This macroreentrant arrhythmias is localized to the right atrium. Atrial flutter can be cured by creating a continuous, transmural, linear lesion between the tricuspid annulus and the inferior vena cava. This procedure is generally referred to as an "isthmus" ablation. Catheter ablation of atrial flutter can be performed with an efficacy greater than 90% and a < 5% risk of recurrence of atrial flutter. Non-isthmus dependant atrial flutter can also be treated with catheter ablation. These regular atrial tachycardias, with rates between 250 and 350 bpm often involve scars created during prior cardiac surgery. The procedure can be accomplished by defining a critical region of conduction which is necessary for maintenance of the arrhythmia. The creation of a linear lesion in this region can result in cure of the tachycardia. Catheter ablation of non-isthmus dependant atrial flutter can be accomplished with an efficacy of greater than 80% and a < 5% risk of major complications.

## Conclusion

Because of the remarkable safety and efficacy of catheter ablation, it is now considered as first line therapy for most types of supraventricular arrhythmias. These include AVNRT, AVRT, atrial tachycardia, and atrial flutter. It is anticipated that the dream of curing atrial fibrillation with ablation techniques will also be realized sometime during the next five years.
